# Gradient differences of immunotherapy efficacy in metastatic melanoma related to sunlight exposure pattern: A population-based study

**DOI:** 10.3389/fonc.2022.1086664

**Published:** 2023-01-05

**Authors:** Mengsong Liu, Wenyuan Li, Xiao Ma, Yuhui Che, Bo Wei, Mulan Chen, Lin Zhong, Siqi Zhao, Anjing Chen, Yaobin Pang, Jinhao Zeng, Jing Guo

**Affiliations:** ^1^ Dermatological Department, Hospital of Chengdu University of Traditional Chinese Medicine, Chengdu, China; ^2^ School of Clinical Medicine, Chengdu University of Traditional Chinese Medicine, Chengdu, China; ^3^ Sichuan Evidence-Based Medicine Center of Traditional Chinese Medicine, Hospital of Chengdu University of Traditional Chinese Medicine, Chengdu, China; ^4^ State Key Laboratory of Southwestern Chinese Medicine Resources, School of Pharmacy, Chengdu University of Traditional Chinese Medicine, Chengdu, China; ^5^ TCM Regulating Metabolic Diseases Key Laboratory of Sichuan Province, Hospital of Chengdu University of Traditional Chinese Medicine, Chengdu, China; ^6^ Department of Geriatrics, Hospital of Chengdu University of Traditional Chinese Medicine, Chengdu, China

**Keywords:** metastatic melanoma, sunlight exposure patterns, SEER database, immune-checkpoint inhibitor (ICIs), epidemiology

## Abstract

**Background:**

Immune checkpoint inhibitors (ICIs) have revolutionized metastatic melanoma (MM) treatment in just a few years. Ultraviolet (UV) in sunlight is the most significant environmental cause of melanoma, which is considered to be the main reason for tumor mutation burden (TMB) increase in melanoma. High TMB usually predicts that PD-1 inhibitors are effective. The sunlight exposure pattern of MM might be a clinical feature that matches TMB. The relationship between sunlight exposure patterns and immunotherapy response in MM is unclear. This study aims to investigate the correlation between sunlight exposure patterns and immunotherapy response in MM and establish nomograms that predict 3- and 5-year overall survival (OS) rate.

**Methods:**

We searched the Surveillance, Epidemiology, and End Results (SEER) database and enrolled MM cases from 2005-2016. According to the advent of ICIs in 2011, the era was divided into the non-ICIs era (2005-2010) and the ICIs era (2011-2016). Patients were divided into three cohorts according to the primary site sunlight exposure patterns: head and neck in the first cohort, trunk arms and legs in the second cohort, and acral sites in the third cohort. We compared survival differences for each cohort between the two eras, performed stratified analysis, established nomograms for predicting 3- and 5-year OS rate, and performed internal validation.

**Results:**

Comparing the survival difference between the ICIs and non-ICIs era, head and neck melanoma showed the greatest improvement in survival, with 3- and 5-year OS rate increasing by 10.2% and 9.1%, respectively (P=0.00011). In trunk arms and legs melanoma, the 3- and 5-year OS rate increased by 4.6% and 3.9%, respectively (P<0.0001). There is no improvement in survival in acral melanoma (AM) between the two eras (P=0.78). The receiver operating characteristic (ROC) curve, area under the ROC curve (AUC) and calibration graphs show good discrimination and accuracy of nomograms. Decision curve analysis (DCA) suggests good clinical utility of nomograms.

**Conclusions:**

Based on the classification of sunlight exposure patterns, there is a gradient difference in immunotherapy efficacy for MM. The degree of sunlight exposure is positively correlated with immunotherapy response. The nomograms are sufficiently accurate to predict 3- and 5-year OS rate for MM, allowing for individualized clinical decisions for future clinical work.

## 1 Introduction

Since ipilimumab was approved by the FDA (U.S. Food and Drug Administration) in 2011, metastatic melanoma (MM) treatments have revolutionized in just a few years (1). Immune checkpoint inhibitors (ICIs) significantly improved overall survival (OS) in MM (2–4). So far, ICIs, such as anti-cytotoxic T lymphocyte-associated protein 4 (anti-CTLA-4) and anti-programmed death protein-1/programmed death-ligand 1 (anti-PD-1/PD-L1), have emerged as the first-line MM treatment regimen.

Ultraviolet (UV) in sunlight is the most significant environmental cause of melanoma (5, 6). The detrimental effects of UV are mainly associated with defective immune surveillance and DNA damage. UV can suppress adaptive immunity and escape tumor immune surveillance by increasing Treg cells and decreasing effector T cells, eventually promoting melanoma development (7). UV irradiation can cause DNA damage, inhibit DNA repair and induce gene mutations (8). Patients with melanoma usually have the strong UV mutation signature (9). C-T conversion in the bipyrimidine moiety is the most common mutation in malignant melanoma cells, mainly caused by UV-induced DNA damage (10). More importantly, UV mutation signatures of primary site are preserved in metastatic sites in MM (11). This suggests that the UV exposure signatures of MM depend on the primary site.

There are significant differences in sunlight exposure between different areas on the human body’s surface. Head and neck generally considered as chronic sunlight exposure areas, trunk arms and legs are intermittent sunlight exposure areas, and acral sites are lesser sunlight exposure areas (12–14). This classification is widely used in clinical practice and showed different immunotherapy responses. Acral melanoma (AM) as a subtype of cutaneous melanoma (CM), has been shown lower ICIs efficacy than other CM (15).

Tumor mutation burden (TMB) has been identified as a promising predictive biomarker for immunotherapy in various cancers (16). High TMB increases the chance that more T cells will recognize neoantigens, leading to clinically relevant better ICIs outcomes (17). MM patients with high TMB have more improvement in OS and progression-free survival (PFS) after receiving ICIs (18, 19). UV exposure is thought to be the main cause of increased TMB in melanoma (20, 21). Dousset et al. measured TMB in chronically sun-exposed areas (face and neck), intermittently sun-exposed areas (trunk arms and legs), and sun-protected areas (mucosal, uveal, foot, toes, etc), found that the values of TMB were 37.2 muts/Mb, 13.6 muts/Mb, and 4 muts/Mb successively, and indicated that TMB was significantly associated with sunlight exposure (20). More importantly, there is a strong concordance between the TMB of primary site and metastatic site cells in MM patients (22). It is consistent with the phenomenon that the UV mutation signature of the primary site is retained at metastasis site. Therefore, we speculated that the sunlight exposure pattern may be a clinical characteristic that matches TMB for MM, causing differences in immunotherapy response.

However, systematic studies on the correlation between sunlight exposure and immunotherapy response in MM are lacking. We hypothesized that immunotherapy efficacy for MM may vary depending on the sunlight exposure pattern at primary site. According to the advent of ICIs in 2011, we divided the era into the non-ICIs era (2005-2010) and the ICIs era (2011-2016). We analyzed immunotherapy efficacy by comparing the survival differences between the two eras and exploring the differences in immunotherapy efficacy between different sunlight exposure pattern cohorts. We also created nomograms to predict 3-and 5-years OS rate in MM patients.

## 2 Materials and methods

### 2.1 Data source

The study is based on the Surveillance, Epidemiology, and End Results (SEER) database. The SEER database has collected survival data of about 34.6% from the United States population cancer registry. It is one of the most authoritative sources of tumor information in the United States. According to the exemption regulations released by the SEER database, the use of the data does not require informed patient consent. SEER database does not contain personally-identifying information.

### 2.2 Patient selection criteria

Research data use agreement was obtained at the SEER program (https://seer.cancer.gov/data). Data was downloaded by using the SEER*Stat software (Version 8.4.0.1) and the SEER-18 registries dataset (November 2019 submission). During 2005-2016, MM patients were selected based on the following criteria: Select melanoma (8720-8799, nevi and melanomas) codes and CM codes in the international classification of diseases for oncology, third edition (ICD-O-3) site/histology. This article only included microscopically confirmed cases, first primary or first only, patients older than or equal to 18 years and surviving more than 0 days. The appropriate codes for MM (stage III and IV) were selected according to Derived AJCC Stage Group 6th Ed (2004-2015), Derived AJCC Stage 7th Ed (2010-2015) and Derived SEER Cmb Stg Grp (2016+).

Data were filtered using the following exclusion criteria: This article excluded primary site ambiguous (“C44.9 Skin, NOS” and “C44.8 Overlapping lesion of skin”), type of reporting source coming from “Autopsy only” and “Death certificate only”, and T0 stage (no evidence of primary tumor).

We collected a large dataset concerning demographic, clinical characteristics and curative treatments. The following variables were evaluated: primary site (head and neck, trunk arms and legs or acral sites); age (18–50 years, 51–70 years or >71 years); sex (male or female); race (white or others); marital status (married or others); T stage (T1, T2, T3 or T4); N stage (N0, N1, N2 or N3); M stage (M0 or M1); ulceration (yes or no); distant organ metastasis (yes or no); surgical margin of primary lesion (narrow, wide or others); local lymph node resection (yes, no or others); metastasectomy (yes or no); radiotherapy (yes or no); chemotherapy (yes or no); vital status (alive or dead); patient ID.

Distant organ metastasis only included bone, brain, liver and lung. Among the surgical margin of primary lesion variables, margins less than or equal to 1 cm were considered narrow margins, and vice versa. Without conventional surgery (such as laser ablation, photodynamic therapy, electrocautery and cryosurgery) and without surgery were defined as others. Among the local lymph node resection variables, needle aspirate biopsy of regional lymph nodes was performed but not known to have been removed was defined as others.

**Figure 1:**
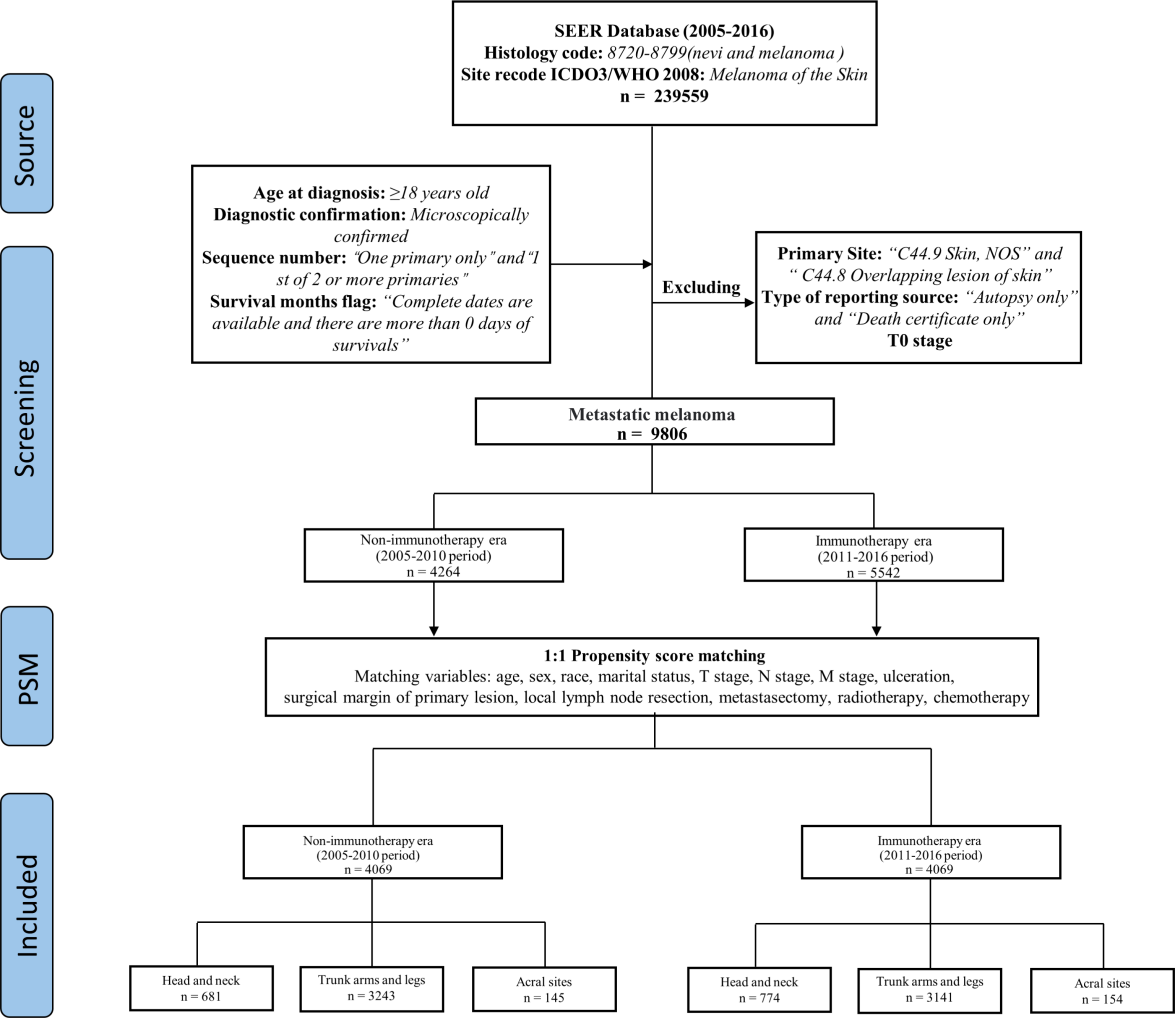
Detailed description of inclusion and exclusion criteria.

We took 2011 as the dividing line, 2005-2010 as the non-ICIs era, and 2011-2016 as the ICIs era. MM patients were divided into three cohorts based on the sunlight exposure patterns of primary site, patients with primary site of head and neck in the first cohort, trunk arms and legs in the second cohort, and acral sites in the third cohort (**Figure 1**).

### 2.3 Propensity score matching

We screened 9806 MM patients, 4264 cases of which were recorded in the non-ICIs era, and 5542 cases were recorded in the ICIs era. Comparing the differences in the variables between the two eras, there were statistically significant differences in surgical margin of primary lesion (P < 0.001, P=0.001) and local lymph node resection in the three cohorts (P=0.026, P < 0.001, P=0.001). The detailed results are shown in **Table 1**.

**Table 1:** Baseline characteristics of metastatic melanoma patients.

	Head and neck			Trunk arms and legs			Acral sites		
	Non-ICIS era (N=711)	ICIS era (N=1021)	P-value	Non-ICIS era (N=3402)	ICIS era (N=4302)	P-value	Non-ICIS era (N=151)	ICIS era (N=219)	P-value
Age									
18-50	192(27.0%)	227(22.2%)	0.041	1240(36.4%)	1247(29.0%)	<0.001	33(21.9%)	47(21.5%)	0.995
51-70	317(44.6%)	461(45.2%)		1448(42.6%)	2164(50.3%)		71(47.0%)	103(47.0%)	
>70	202(28.4%)	333(32.6%)		714(21.0%)	891(20.7%)		47(31.1%)	69(31.5%)	
Sex									
Male	544(76.5%)	792(77.6%)	0.647	2119(62.3%)	2622(60.9%)	0.24	82(54.3%)	119(54.3%)	1
Female	167(23.5%)	229(22.4%)		1283(37.7%)	1680(39.1%)		69(45.7%)	100(45.7%)	
Race									
White	691(97.2%)	991(97.1%)	0.994	3293(96.8%)	4155(96.6%)	0.65	119(78.8%)	172(78.5%)	1
Others	20(2.8%)	30(2.9%)		109(3.2%)	147(3.4%)		32(21.2%)	47(21.5%)	
Marital status									
Married	418(58.8%)	606(59.4%)	0.853	1979(58.2%)	2371(55.1%)	0.008	104(68.9%)	123(56.2%)	0.018
Others	293(41.2%)	415(40.6%)		1423(41.8%)	1931(44.9%)		47(31.1%)	96(43.8%)	
T stage									
T1	107(15.0%)	145(14.2%)	0.816	512(15.1%)	584(13.6%)	0.018	17(11.3%)	22(10.0%)	0.976
T2	173(24.3%)	240(23.5%)		889(26.1%)	1105(25.7%)		21(13.9%)	33(15.1%)	
T3	193(27.1%)	272(26.6%)		982(28.9%)	1188(27.6%)		51(33.8%)	74(33.8%)	
T4	238(33.5%)	364(35.7%)		1019(30.0%)	1425(33.1%)		62(41.1%)	90(41.1%)	
N stage									
N0	54(7.6%)	95(9.3%)	0.178	145(4.3%)	163(3.8%)	0.382	6(4.0%)	5(2.3%)	0.605
N1	322(45.3%)	414(40.5%)		1867(54.9%)	2437(56.6%)		72(47.7%)	98(44.7%)	
N2	206(29.0%)	327(32.0%)		895(26.3%)	1107(25.7%)		43(28.5%)	74(33.8%)	
N3	129(18.1%)	185(18.1%)		495(14.6%)	595(13.8%)		30(19.9%)	42(19.2%)	
M stage									
M0	587(82.6%)	827(81.0%)	0.446	3006(88.4%)	3838(89.2%)	0.252	136(90.1%)	201(91.8%)	0.702
M1	124(17.4%)	194(19.0%)		396(11.6%)	464(10.8%)		15(9.9%)	18(8.2%)	
Ulceration									
Yes	299(42.1%)	471(46.1%)	0.103	1599(47.0%)	2048(47.6%)	0.614	102(67.5%)	140(63.9%)	0.543
No	412(57.9%)	550(53.9%)		1803(53.0%)	2254(52.4%)		49(32.5%)	79(36.1%)	
Distant organ metastasis									
Yes		136(13.3%)			331(7.7%)			14(6.4%)	
No		885(86.7%)			3971(92.3%)			205(93.6%)	
Surgical margin of primary lesion									
Narrow	250(35.2%)	442(43.3%)	<0.001	976(28.7%)	1595(37.1%)	<0.001	31(20.5%)	83(37.9%)	0.001
Wide	361(50.8%)	406(39.8%)		2021(59.4%)	2186(50.8%)		78(51.7%)	94(42.9%)	
Others	100(14.1%)	173(16.9%)		405(11.9%)	521(12.1%)		42(27.8%)	42(19.2%)	
Local lymph node resection									
Yes	482(67.8%)	630(61.7%)	0.026	2396(70.4%)	2811(65.3%)	<0.001	116(76.8%)	136(62.1%)	0.001
No	118(16.6%)	189(18.5%)		333(9.8%)	410(9.5%)		10(6.6%)	10(4.6%)	
Others	111(15.6%)	202(19.8%)		673(19.8%)	1081(25.1%)		25(16.6%)	73(33.3%)	
Metastasectomy									
Yes	109(15.3%)	139(13.6%)	0.351	226(6.6%)	285(6.6%)	1	13(8.6%)	10(4.6%)	0.173
No	602(84.7%)	882(86.4%)		3176(93.4%)	4017(93.4%)		138(91.4%)	209(95.4%)	
Radiotherapy									
Yes	155(21.8%)	193(18.9%)	0.156	241(7.1%)	278(6.5%)	0.3	10(6.6%)	13(5.9%)	0.96
No	556(78.2%)	828(81.1%)		3161(92.9%)	4024(93.5%)		141(93.4%)	206(94.1%)	
Chemotherapy									
Yes	95(13.4%)	124(12.1%)	0.499	412(12.1%)	423(9.8%)	0.002	16(10.6%)	18(8.2%)	0.552
No	616(86.6%)	897(87.9%)		2990(87.9%)	3879(90.2%)		135(89.4%)	201(91.8%)	

Propensity score matching (PSM) analysis was performed to reduce the bias. Except for distant organ metastasis, as it only contains data from the ICIs era. The patients were matched 1:1 by estimated propensity scores with a caliper width of 0.02. After matching, 4069 matched pairs (8138 cases) were included in the analysis (**Figure 1**), and the variables were almost balanced between the two eras shown in **Table 2**. This paper uses propensity score matching data for analysis and evaluation.

**Table 2:** Baseline characteristics of metastatic melanoma patients after PSM.

	Head and neck			Trunk arms and legs			Acral sites		
	Non-ICIS era (N=681)	ICIS era (N=774)	P-value	Non-ICIS era (N=3243)	ICIS era (N=3141)	P-value	Non-ICIS era (N=145)	ICIS era (N=154)	P-value
Age									
18-50	170(25.0%)	193(24.9%)	0.104	1101(34.0%)	1001(31.9%)	0.056	28(19.3%)	41(26.6%)	0.305
51-70	314(46.1%)	320(41.3%)		1437(44.3%)	1485(47.3%)		71(49.0%)	66(42.9%)	
>70	197(28.9%)	261(33.7%)		705(21.7%)	655(20.9%)		46(31.7%)	47(30.5%)	
Sex									
Male	519(76.2%)	596(77.0%)	0.769	2023(62.4%)	1967(62.6%)	0.861	77(53.1%)	84(54.5%)	0.893
Female	162(23.8%)	178(23.0%)		1220(37.6%)	1174(37.4%)		68(46.9%)	70(45.5%)	
Race									
White	662(97.2%)	750(96.9%)	0.846	3140(96.8%)	3019(96.1%)	0.143	113(77.9%)	117(76.0%)	0.792
Others	19(2.8%)	24(3.1%)		103(3.2%)	122(3.9%)		32(22.1%)	37(24.0%)	
Marital status									
Married	396(58.1%)	461(59.6%)	0.622	1861(57.4%)	1734(55.2%)	0.084	98(67.6%)	86(55.8%)	0.049
Others	285(41.9%)	313(40.4%)		1382(42.6%)	1407(44.8%)		47(32.4%)	68(44.2%)	
T stage									
T1	102(15.0%)	115(14.9%)	0.713	473(14.6%)	436(13.9%)	0.8	17(11.7%)	16(10.4%)	0.941
T2	166(24.4%)	175(22.6%)		858(26.5%)	818(26.0%)		20(13.8%)	20(13.0%)	
T3	179(26.3%)	223(28.8%)		925(28.5%)	910(29.0%)		49(33.8%)	57(37.0%)	
T4	234(34.4%)	261(33.7%)		987(30.4%)	977(31.1%)		59(40.7%)	61(39.6%)	
N stage									
N0	53(7.8%)	73(9.4%)	0.177	139(4.3%)	120(3.8%)	0.404	6(4.1%)	4(2.6%)	0.631
N1	308(45.2%)	308(39.8%)		1780(54.9%)	1773(56.4%)		70(48.3%)	66(42.9%)	
N2	199(29.2%)	251(32.4%)		857(26.4%)	830(26.4%)		43(29.7%)	53(34.4%)	
N3	121(17.8%)	142(18.3%)		467(14.4%)	418(13.3%)		26(17.9%)	31(20.1%)	
M stage									
M0	563(82.7%)	616(79.6%)	0.152	2873(88.6%)	2779(88.5%)	0.916	131(90.3%)	140(90.9%)	1
M1	118(17.3%)	158(20.4%)		370(11.4%)	362(11.5%)		14(9.7%)	14(9.1%)	
Ulceration									
Yes	288(42.3%)	373(48.2%)	0.028	1544(47.6%)	1575(50.1%)	0.046	99(68.3%)	107(69.5%)	0.92
No	393(57.7%)	401(51.8%)		1699(52.4%)	1566(49.9%)		46(31.7%)	47(30.5%)	
Distant organ metastasis									
Yes		110(14.2%)			261(8.3%)			10(6.5%)	
No		664(85.8%)			2880(91.7%)			144(93.5%)	
Surgical margin of primary lesion									
Narrow	249(36.6%)	283(36.6%)	0.267	972(30.0%)	861(27.4%)	0.078	31(21.4%)	48(31.2%)	0.105
Wide	335(49.2%)	358(46.3%)		1873(57.8%)	1879(59.8%)		73(50.3%)	74(48.1%)	
Others	97(14.2%)	133(17.2%)		398(12.3%)	401(12.8%)		41(28.3%)	32(20.8%)	
Local lymph node resection									
Yes	457(67.1%)	488(63.0%)	0.106	2252(69.4%)	2155(68.6%)	0.760	110(75.9%)	105(68.2%)	0.139
No	114(16.7%)	163(21.1%)		322(9.9%)	324(10.3%)		10(6.9%)	8(5.2%)	
Others	110(16.2%)	123(15.9%)		669(20.6%)	662(21.1%)		25(17.2%)	41(26.6%)	
Metastasectomy									
Yes	105(15.4%)	109(14.1%)	0.520	214(6.6%)	228(7.3%)	0.323	12(8.3%)	6(3.9%)	0.178
No	576(84.6%)	665(85.9%)		3029(93.4%)	2913(92.7%)		133(91.7%)	148(96.1%)	
Radiotherapy									
Yes	145(21.3%)	169(21.8%)	0.852	218(6.7%)	228(7.3%)	0.428	8(5.5%)	11(7.1%)	0.735
No	536(78.7%)	605(78.2%)		3025(93.3%)	2913(92.7%)		137(94.5%)	143(92.9%)	
Chemotherapy									
Yes	88(121.9%)	111(14.3%)	0.478	374(11.5%)	375(11.9%)	0.642	12(8.3%)	17(11.0%)	0.541
No	593(87.1%)	663(85.7%)		2869(88.5%)	2766(88.1%)		133(91.7%)	137(89.0%)	

### 2.4 Construction and validation of prognostic nomograms

Univariable and multivariable Cox analyses were used to screen independent prognostic factors in the training cohorts at P<0.05 level. Independent prognostic factors as variables were used to create nomograms. Nomograms were used to predict 3- and 5-year OS rate. Evaluate nomograms performance by receiver operating characteristic (ROC) curve, area under the ROC curve (AUC), calibration graph and decision curve analysis (DCA). The discriminative power of nomograms was assessed by using the ROC curve and AUC. The accuracy of nomograms was evaluated by using calibration graph. The ROC curve reflects the specificity and sensitivity of the prediction model (23). The closer the ROC curve is to the upper left corner of the graph, the higher the accuracy of the prediction model (24). Bootstrap resampling (1000 resampling) was used for the calibration graph to determine the calibration capability of nomograms. The 45° line represents perfect calibration, the closer the better. The clinical utility of nomograms was evaluated by DCA. It is a method to determine whether use of the prediction model to inform clinic decision-making would do more good than harm (25). It is used to derive the net benefit of the prediction model across different threshold probabilities (26). Survival rate for high- and low-risk cases were compared by using Kaplan-Meier (KM) survival curves and log-rank test.

### 2.5 Statistical analysis

All statistical analyses were performed by R studio version 4.1.3. Statistical significance was set at P < 0.05 level. Continuous variables were expressed as mean ± standard deviation, Mann-Whitney U test was used for comparison. χ^2^ test or Fisher’s exact test was used for categorical variable comparison. 3- and 5-year OS rate were compared by KM survival curves and log-rank test. Univariable and multivariable Cox analyses were used to screen for independent prognostic factors. Nomograms were created by using the rms package in R Studio (http://www.r-project.org/).

## 3 Results

### 3.1 Gradient differences in immunotherapy efficacy among three cohorts

**Figure 2:**
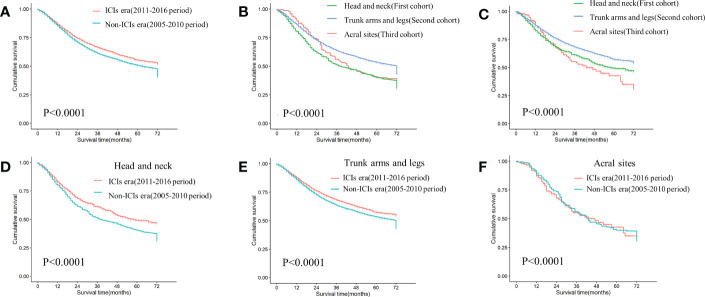
KM curves of differences in survival among cohorts **(A)** Comparing survival differences between non-ICIs era and ICIs era (P<0.0001). **(B)** Differences in survival among three cohorts in the non-ICIs era (P<0.0001). **(C)** Differences in survival among three cohorts in the ICIs era (P<0.0001). **(D)** Differences in survival between the non-ICIs era and the ICIs era in the first cohort (P=0.00011). **(E)** Differences in survival between the non-ICIs era and the ICIs era in the second cohort (P<0.0001). **(F)** Differences in survival between the non-ICIs era and the ICIs era in the third cohort (P=0.78).

Survival was significantly improved in the ICIs era (**Figure 2A**).In the non-ICIs and ICIs era, there was a significant survival gap between the first (head and neck) and second (trunk, arms and legs) cohorts. Survival rate was consistently higher in the second cohort than in the first. In the non-ICIs era, when survival was longer than 24 months, the second cohort had better survival outcomes than the third cohort (acral sites) (**Figure 2B**). In the ICIs era, survival rate increased significantly in the first and second cohorts. The survival gap between the third cohort (acral sites) and the other cohorts had been widened. When survival was less than 24 months, the third cohort (acral sites) had the lowest survival rate (**Figure 2C**).

There was a graded difference in ICIs efficacy when comparing 3-year and 5-year OS rate in three cohorts between the two eras. The first cohort benefited the most from ICIs (P=0.00011), with 3-year and 5-year OS rate increasing by 10.2% and 9.1%, respectively (**Figure 2D**). In the second cohort, the 3-year and 5-year OS rate increased by 4.6% and 3.9%, respectively (P<0.0001) (**Figure 2E**). However, there was no survival difference between the two eras in the third cohort (P=0.78) (**Figure 2F**).

### 3.2 Stratified analysis of cohorts in the ICIs era

**Figure 3:**
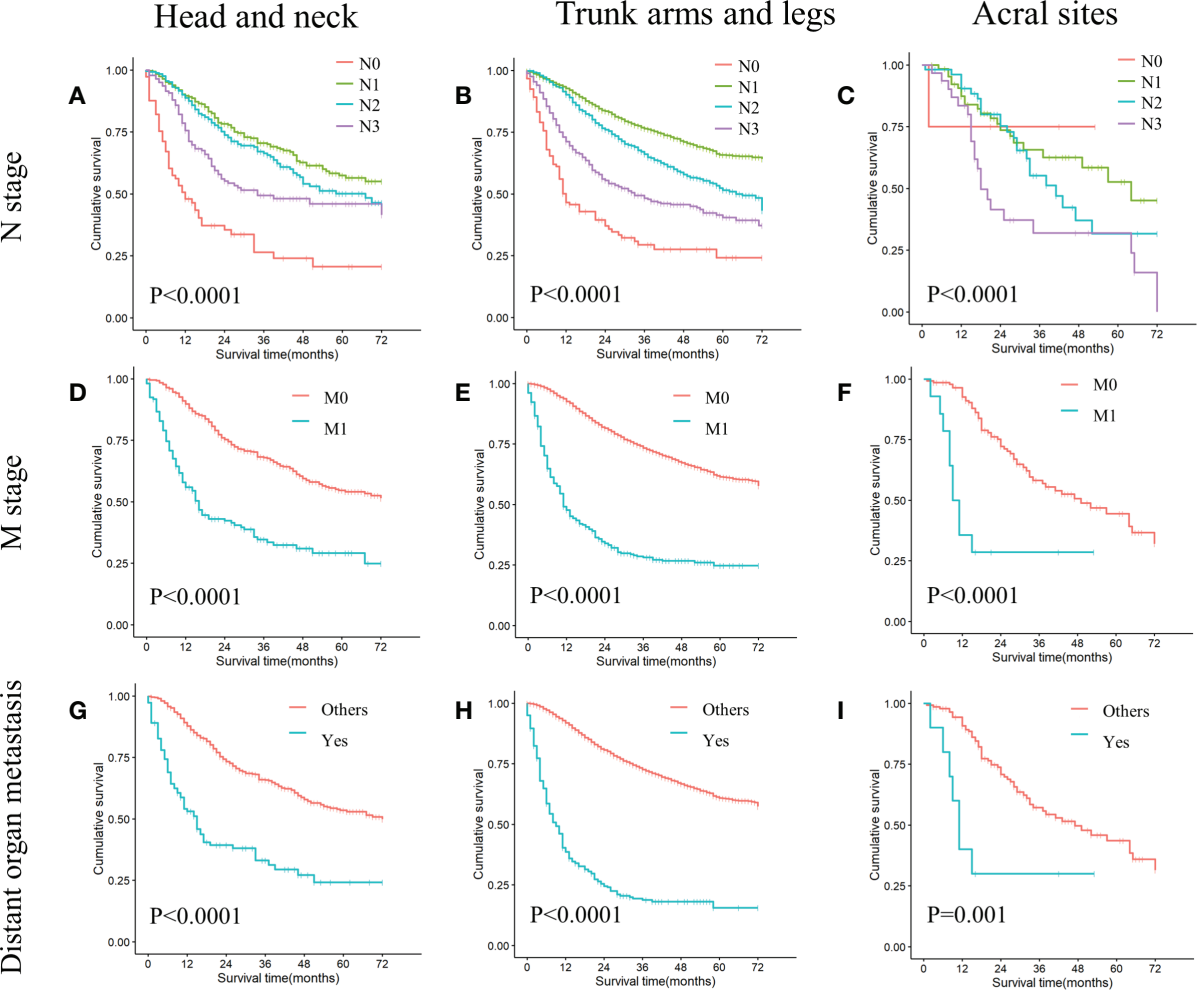
KM curves of stratified analysis in the ICIs era: N stage, M stage, distant organ metastasis. **(A)** Comparing survival differences in N stage in head and neck melanoma (P<0.0001). **(B)** Comparing survival differences in N stage in trunk arms and legs melanoma (P<0.0001). **(C)** Comparing survival differences in N stage in acral melanoma (P<0.0001). **(D)** Comparing survival differences in M stage in head and neck melanoma (P<0.0001). **(E)** Comparing survival differences in M stage in trunk arms and legs melanoma (P<0.0001). **(F)** Comparing survival differences in M stage in acral melanoma (P<0.0001). **(G)** Comparing survival differences in distant organ metastasis in head and neck melanoma (P<0.0001). **(H)** Comparing survival differences in distant organ metastasis in trunk arms and legs melanoma (P<0.0001). **(I)** Comparing survival differences in distant organ metastasis in acral melanoma (P<0.0001).

In all cohorts, patients with N1 stage, M0 stage, and no distant organ metastases showed better survival (**Figure 3**) (**Table 3**). In addition, patients with non-acral CM (the first and second cohorts) who had T2 stage, wide surgical margin of primary lesion (>1cm), and local lymph node resection had higher OS rate (**Table 3**).

**Table 3:** Stratified analysis of cohorts in the ICIS era.

	Head and neck		Trunk arms and legs		Acral sites	
	Median (months)	P-value	Median (months)	P-value	Median (months)	P-value
Age						
18-50	20.0		22.0		20.0	
51-70	18.0		19.0		24.0	
>70	14.0	0.479	16.0	0.004	21.0	0.299
Sex						
Male	17.5		18.0		21.0	
Female	17.0	0.336	18.0	0.705	23.0	0.424
Race						
White	18.0		18.0		21.0	
Others	13.0	0.848	19.0	0.545	22.5	0.510
Marital status						
Married	16.0		19.0		18.0	
Others	18.0	0.432	18.0	0.020	24.0	0.957
T stage						
T1	17.0		18.0		16.5	
T2	21.0		22.5		21.0	
T3	20.0		22.0		22.0	
T4	13.0	0.023	15.0	0.000	24.0	0.932
N stage						
N0	8.0		10.0		2.0	
N1	21.0		20.0		21.0	
N2	19.0		21.0		27.0	
N3	14.0	0.000	13.0	0.000	16.5	0.000
M stage						
M0	19.0		20.0		24.0	
M1	9.0	0.000	9.5	0.000	6.5	0.000
Ulceration						
Yes	16.0		16.0		19.0	
No	18.0	0.044	22.0	0.000	24.0	0.684
Distant organ metastasis						
Yes	6.0		6.0		2.0	
No	18.0	0.000	19.0	0.000	22.0	0.000
Surgical margin of primary lesion						
Narrow	18.0		18.0		21.0	
Wide	20.0		21.0		18.0	
Others	9.0	0.000	11.5	0.000	24.0	0.670
Local lymph node resection						
Yes	20.0		20.0		20.5	
No	9.5		11.0		9.0	
Others	15.5	0.000	18.0	0.000	26.5	0.751
Metastasectomy						
Yes	15.0		20.5		26.5	
No	18.0	0.960	18.0	0.828	21.0	0.858
Radiotherapy						
Yes	16.0		13.0		21.5	
No	18.0	0.587	19.0	0.000	21.0	0.172
Chemotherapy						
Yes	14.0		15.0		19.5	
No	18.0	0.799	19.0	0.001	23.0	0.983

### 3.3 Univariable and multivariable cox regression analysis in the ICIs era

In the univariable analysis, M0 stage, no distant organ metastasis, routine surgery at the primary site, no radiotherapy and chemotherapy showed significant positive survival benefits in all cohorts of the ICIs era. Multivariable analyses of the first and second cohorts showed a significant reduction in survival among patients older than 70 years, with ulcers, and without routine surgery at the primary site. Only female showed positive survival in the first (head and neck) and second (trunk, arms and legs) cohorts. Age, sex, ulceration, and surgical margin of primary lesion were independent prognostic factors in non-acral CM (the first and second cohorts). M stage and radiotherapy were independent prognostic factors in AM (the third cohort) (**Table 4**).

**Table 4:** Univariate and multivariate cox analysis of all cohorts in the ICIS era.

	Head and neck				Trunk arms and legs				Acral sites			
	UnivariateHR (CI)	P-value	MultivariateHR (CI)	P-value	UnivariateHR (CI)	P-value	MultivariateHR (CI)	P-value	UnivariateHR (CI)	P-value	MultivariateHR (CI)	P-value
Age												
18-50												
51-70	1.38 (1-1.9)	0.05	1.32(0.95-1.84)	0.09	1.59(1.36-1.87)	0.00	1.47(1.25-1.72)	0.00	1.35(0.7-2.58)	0.37		
>70	2.56(1.87-3.5)	0.00	2.15(1.54-2.99)	0.00	3.03(2.56-3.58)	0.00	2.44(2.05-2.9)	0.00	1.54(0.78-3.02)	0.21		
Sex												
Male												
Female	0.74(0.56-0.98)	0.04	0.73(0.55-0.98)	0.03	0.69(0.6-0.78)	0.00	0.77(0.67-0.88)	0.00	0.72(0.44-1.17)	0.19		
Race												
Others												
White	1.08(0.54-2.18)	0.83			0.79(0.59-1.05)	0.11			0.61(0.37-1.02)	0.06		
Marital status												
Others												
Married	0.83(0.66-1.03)	0.09			0.72(0.64-0.81)	0.00	0.81(0.71-0.91)	0.00	0.78(0.48-1.25)	0.30		
T stage												
T1												
T2	0.62(0.42-0.93)	0.02	0.81(0.54-1.21)	0.31	0.7(0.55-0.89)	0.00	0.84(0.66-1.07)	0.16	0.71(0.28-1.8)	0.47		
T3	0.91(0.64-1.31)	0.61	0.98(0.68-1.43)	0.92	1.23(1-1.52)	0.05	0.97(0.78-1.2)	0.76	0.54(0.24-1.18)	0.12		
T4	1.56(1.12-2.18)	0.01	1.43(1.01-2.05)	0.05	2.31(1.89-2.82)	0.00	1.31(1.06-1.61)	0.01	0.87(0.41-1.84)	0.72		
N stage												
N0												
N1	0.24(0.17-0.34)	0.00	0.56(0.36-0.88)	0.01	0.19(0.15-0.24)	0.00	0.77(0.58-1.03)	0.08	1.35(0.18-10.07)	0.77		
N2	0.29(0.21-0.41)	0.00	0.56(0.35-0.88)	0.01	0.3(0.24-0.39)	0.00	1.04(0.78-1.4)	0.77	1.7(0.23-12.59)	0.60		
N3	0.45(0.31-0.64)	0.00	0.9(0.56-1.46)	0.68	0.52(0.41-0.68)	0.00	1.34(1-1.79)	0.05	3.46(0.46-25.82)	0.23		
M stage												
M0												
M1	2.97(2.34-3.76)	0.00	1.45(0.94-2.25)	0.09	4.73(4.1-5.45)	0.00	1.13(0.82-1.57)	0.46	4.34(2.19-8.59)	0.00	7.33(2.02-26.54)	0.00
Ulceration												
No												
Yes	1.71(1.37-2.14)	0.00	1.44(1.14-1.84)	0.00	2.6(2.29-2.96)	0.00	2.08(1.81-2.39)	0.00	1.31(0.75-2.27)	0.34		
Distant organ metastasis												
No												
Yes	3.16(2.43-4.12)	0.00	1.17(0.74-1.84)	0.49	6.49(5.56-7.58)	0.00	2.77(2.01-3.83)	0.00	3.49(1.58-7.7)	0.00	0.32(0.07-1.34)	0.12
Surgical margin of primary lesion												
Narrow												
Wide	0.87(0.68-1.13)	0.30	0.87(0.67-1.13)	0.30	1.1(0.95-1.29)	0.20	1.11(0.95-1.3)	0.18	1.28(0.7-2.33)	0.42	1.17(0.63-2.16)	0.61
Others	2.22(1.66-2.96)	0.00	1.45(1.04-2.02)	0.03	3.17(2.64-3.8)	0.00	1.56(1.27-1.92)	0.00	2.18(1.12-4.25)	0.02	1.46(0.68-3.11)	0.33
Local lymph node resection												
No												
Yes	0.4(0.31-0.51)	0.00	0.77(0.55-1.09)	0.14	0.24(0.21-0.28)	0.00	0.7(0.56-0.86)	0.00	0.55(0.22-1.39)	0.21		
Others	0.34(0.23-0.49)	0.00	0.69(0.45-1.06)	0.09	0.24(0.19-0.29)	0.00	0.66(0.52-0.84)	0.00	0.58(0.21-1.58)	0.29		
Metastasectomy												
No												
Yes	1.13(0.83-1.54)	0.43			1.09(0.87-1.36)	0.47			3.43(1.05-11.19)	0.04	2.47(0.68-9)	0.17
Radiotherapy												
No												
Yes	1.64(1.29-2.08)	0.00	1.26(0.98-1.62)	0.07	3.68(3.11-4.35)	0.00	1.41(1.17-1.71)	0.00	2.63(1.3-5.35)	0.01	2.6(1.21-5.58)	0.01
Chemotherapy												
No												
Yes	1.35(1.01-1.8)	0.04	1.08(0.79-1.47)	0.64	1.53(1.3-1.8)	0.00	1.11(0.93-1.32)	0.24	1.99(1.04-3.82)	0.04	1.55(0.78-3.1)	0.21

### 3.4 Prognostic nomograms of metastatic melanoma

We include data from two eras to create nomograms and set the era as a new variable. The ICIs era as yes (assuming to receive ICIs therapy) and the non-ICIs era as no (not receiving ICIs therapy). All used a 2-to-1 ratio to randomly assign cases to the training set for nomogram construction and the validation set used to perform internal validation. We use data from the training set to select variables by univariable and multivariable Cox regression analysis at P<0.05 level (**Table 5**). Multivariable Cox proportional hazards models were built to create nomograms.

**Table 5:** Univariate and multivariate cox analysis of training sets.

	Head and neck				Trunk arms and legs				Acral sites			
	UnivariateHR (CI)	P-value	MultivariateHR (CI)	P-value	UnivariateHR (CI)	P-value	MultivariateHR (CI)	P-value	UnivariateHR (CI)	P-value	MultivariateHR (CI)	P-value
Age												
18-50												
51-70	1.77(1.37-2.29)	0.00	1.81(1.39-2.36)	0.00	1.71(1.52-1.92)	0.00	1.51(1.35-1.7)	0.00	1.44(0.81-2.56)	0.22	1.2(0.66-2.19)	0.54
>70	2.94(2.26-3.83)	0.00	2.96(2.24-3.91)	0.00	3.22(2.85-3.65)	0.00	2.83(2.5-3.22)	0.00	2.05(1.13-3.71)	0.02	1.94(1.03-3.64)	0.04
Sex												
Male												
Female	0.83(0.67-1.02)	0.07			0.7(0.64-0.77)	0.00	0.74(0.67-0.82)	0.00	0.73(0.5-1.08)	0.11		
Race												
Others												
White	1.25(0.71-2.22)	0.44			0.76(0.61-0.95)	0.02	0.9(0.72-1.13)	0.37	0.57(0.38-0.87)	0.01	0.59(0.38-0.9)	0.02
Marital status												
Others												
Married	0.76(0.64-0.9)	0.00	0.8(0.67-0.96)	0.02	0.77(0.7-0.84)	0.00	0.86(0.79-0.95)	0.00	0.76(0.51-1.12)	0.16		
T stage												
T1												
T2	0.75(0.55-1.01)	0.06	0.87(0.64-1.18)	0.36	0.79(0.66-0.93)	0.01	0.88(0.74-1.05)	0.16	0.61(0.27-1.37)	0.23		
T3	1(0.75-1.33)	1.00	1.07(0.8-1.43)	0.65	1.3(1.11-1.52)	0.00	1.06(0.9-1.24)	0.50	0.65(0.34-1.22)	0.18		
T4	1.39(1.06-1.82)	0.02	1.26(0.95-1.66)	0.11	2.34(2.02-2.72)	0.00	1.41(1.2-1.64)	0.00	1.34(0.74-2.41)	0.34		
N stage												
N0												
N1	0.31(0.23-0.4)	0.00	0.73(0.5-1.06)	0.10	0.27(0.22-0.32)	0.00	1.3(1.03-1.64)	0.03	0.67(0.24-1.88)	0.45		
N2	0.39(0.29-0.51)	0.00	0.8(0.55-1.18)	0.26	0.39(0.32-0.47)	0.00	1.62(1.27-2.06)	0.00	0.93(0.33-2.64)	0.90		
N3	0.57(0.42-0.76)	0.00	1.31(0.88-1.96)	0.18	0.73(0.6-0.9)	0.00	2.4(1.89-3.06)	0.00	1.56(0.55-4.43)	0.41		
M stage												
M0												
M1	2.54(2.09-3.09)	0.00	1.44(1.05-1.99)	0.03	4.3(3.83-4.82)	0.00	2.08(1.74-2.5)	0.00	2.86(1.67-4.88)	0.00	1.88(0.93-3.82)	0.08
Ulceration												
No												
Yes	1.65(1.39-1.97)	0.00	1.33(1.11-1.6)	0.00	2.25(2.05-2.47)	0.00	1.77(1.6-1.95)	0.00	2.4(1.47-3.91)	0.00	2.53(1.49-4.31)	0.00
Distant organ metastasis												
No												
Yes	2.31(1.77-3.01)	0.00	1.51(1.05-2.17)	0.03	5.73(4.88-6.72)	0.00	2.02(1.62-2.5)	0.00	4.15(1.51-11.41)	0.01	2.71(0.82-8.9)	0.10
The surgical margin of primary lesion												
Narrow												
Wide	0.95(0.78-1.15)	0.57	0.99(0.81-1.21)	0.91	0.95(0.85-1.05)	0.30	0.95(0.85-1.06)	0.35	1.07(0.65-1.76)	0.80	1(0.6-1.66)	0.98
Others	1.92(1.51-2.44)	0.00	1.58(1.2-2.07)	0.00	2.41(2.11-2.76)	0.00	1.34(1.16-1.56)	0.00	1.77(1.03-3.03)	0.04	1.34(0.74-2.44)	0.34
Local lymph node resection												
No												
Yes	0.44(0.36-0.54)	0.00	0.88(0.67-1.17)	0.39	0.31(0.28-0.36)	0.00	0.77(0.66-0.91)	0.00	0.66(0.27-1.63)	0.37		
Others	0.39(0.3-0.52)	0.00	0.75(0.55-1.04)	0.08	0.29(0.25-0.34)	0.00	0.7(0.59-0.84)	0.00	0.83(0.32-2.17)	0.70		
Metastasectomy												
No												
Yes	1.13(0.89-1.42)	0.31			1.07(0.9-1.28)	0.44			2.23(1.12-4.43)	0.02	2.08(0.93-4.62)	0.07
Radiotherapy												
No												
Yes	1.32(1.08-1.61)	0.01	1.01(0.82-1.25)	0.92	3.45(3.01-3.97)	0.00	1.64(1.41-1.9)	0.00	2.54(1.32-4.89)	0.01	2.72(1.38-5.39)	0.00
Chemotherapy												
No												
Yes	1.46(1.15-1.85)	0.00	1.09(0.84-1.42)	0.51	1.46(1.29-1.66)	0.00	1.07(0.94-1.23)	0.31	1.35(0.77-2.37)	0.30		
ICIS therapy												
No												
Yes	0.73(0.61-0.87)	0.00	0.62(0.51-0.75)	0.00	0.84(0.76-0.92)	0.00	0.76(0.68-0.83)	0.00	1.13(0.76-1.68)	0.54		

**Figure 4:**
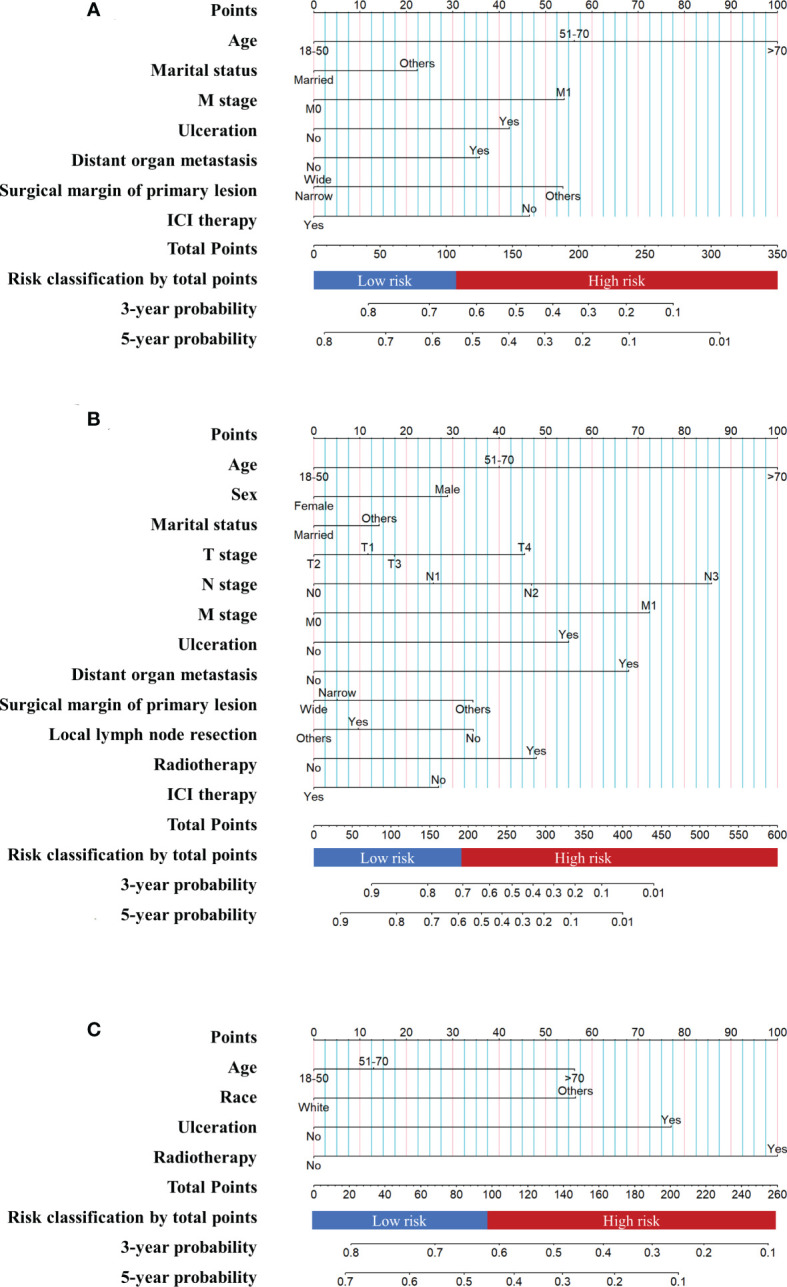
Prognostic nomograms of metastatic melanoma **(A)** The nomogram of the first cohort. **(B)** The nomogram of the second cohort. **(C)** The nomogram of the third cohort.

**Figure 5:**
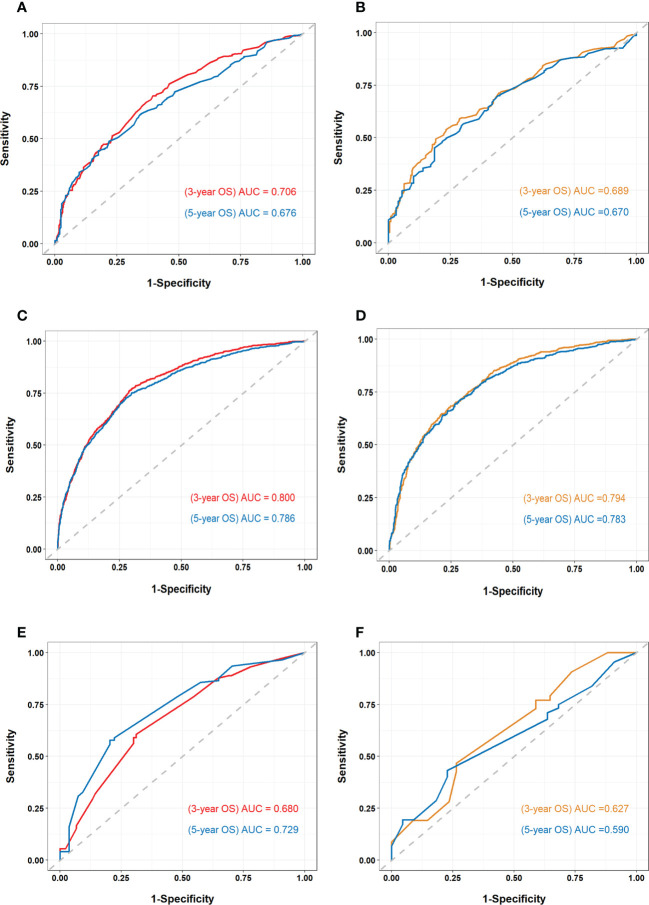
Receiver operating characteristic (ROC) curve and area under the ROC curve (AUC) of all cohorts **(A)** ROC curve and AUC of the first cohort training set. **(B)** ROC curve and AUC of the first cohort validation set. **(C)** ROC curve and AUC of the second cohort training set. **(D)** ROC curve and AUC of the second cohort validation set. **(E)** ROC curve and AUC of the third cohort training set. **(F)** ROC curve and AUC of the third cohort validation set.

The nomogram of the first cohort is constructed by age, marital status, M stage, ulceration, distant organ metastasis, surgical margin of primary lesion and ICIs therapy (**Figure 4A**). The AUC of the training set predicting 3- and 5-year OS rate are 0.706 and 0.676 respectively (**Figure 5A**), and the AUC of the validation set are 0.689 and 0.670, respectively (**Figure 5B**). The nomogram of the second cohort is constructed based on variables such as age, sex, marital status, T stage, N stage, M stage, ulceration, distant organ metastasis, surgical margin of primary lesion, local lymph node resection, radiotherapy and ICIs therapy (**Figure 4B**). The AUC of the training set predicting 3- and 5-year OS rate are 0.800 and 0.786 respectively (**Figure 5C**), and the AUC of the validation set are 0.794 and 0.783, respectively (**Figure 5D**). The nomogram variables for the third cohort consist of age, race, ulceration and radiotherapy (**Figure 4C**). The AUC of the training set predicting 3- and 5-year OS rate are 0.680 and 0.729 respectively (**Figure 5E**), and the AUC of the validation set are 0.627 and 0.590, respectively (**Figure 5F**).

**Figure 6:**
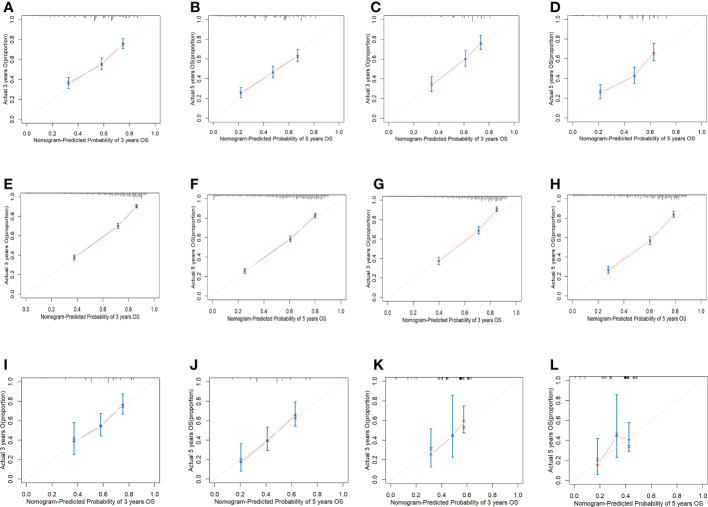
Calibration graphs of all cohorts. **(A)** Calibration graph of predicting 3-year OS for the first cohort training set. **(B)** Calibration graph of predicting 5-year OS for the first cohort training set. **(C)** Calibration graph of predicting 3-year OS for the first cohort validation set. **(D)** Calibration graph of predicting 5-year OS for the first cohort validation set. **(E)** Calibration graph of predicting 3-year OS for the second cohort training set. **(F)** Calibration graph of predicting 5-year OS for the second cohort training set. **(G)** Calibration graph of predicting 3-year OS for the second cohort validation set. **(H)** Calibration graph of predicting 5-year OS for the second cohort validation set. **(I)** Calibration graph of predicting 3-year OS for the third cohort training set. **(J)** Calibration graph of predicting 5-year OS for the third cohort training set. **(K)** Calibration graph of predicting 3-year OS for the third cohort validation set. **(L)** Calibration graph of predicting 5-year OS for the third cohort validation set.

**Figure 7:**
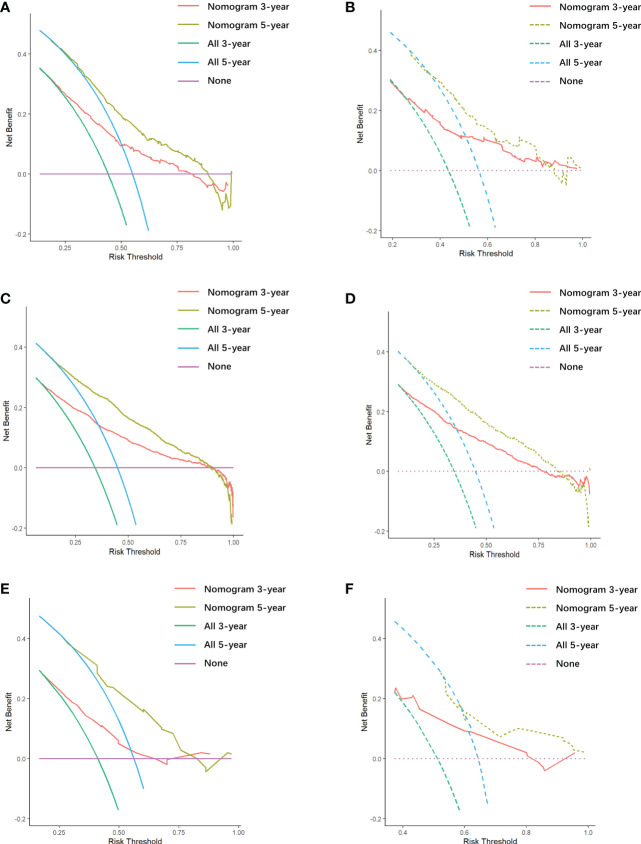
Decision curve analysis (DCA) of all cohorts. **(A)** DCA of the first cohort training set. **(B)** DCA of the first cohort validation set. **(C)** DCA of the second cohort training set. **(D)** DCA of the second cohort validation set. **(E)** DCA of the third cohort training set. **(F)** DCA of the third cohort validation set.

There is appropriate consistency between the observed and predicted probabilities of 3- and 5-year OS rate for the three nomograms, and both training and validation set calibration graphs are close to the 45-degree line (**Figure 6**). The internal validation for the nomograms suggests good predictive consistency. DCA for both the training and validation sets is performed to show the clinical usefulness of the nomogram (**Figure 7**).

### 3.5 Risk classification

**Figure 8:**
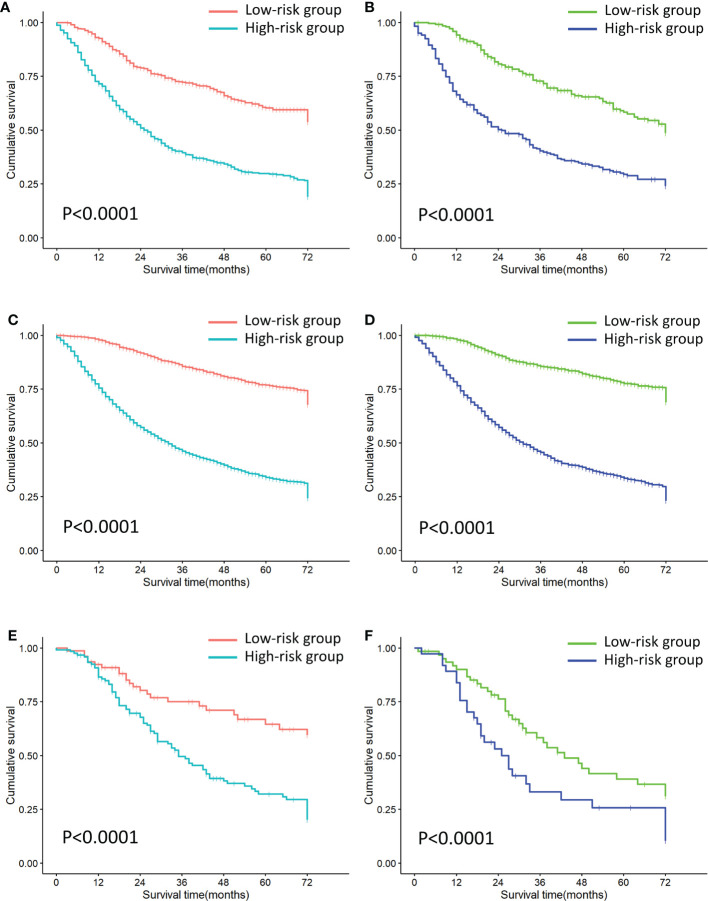
Risk stratification of all cohorts. **(A)** Risk stratification of the first cohort training set. **(B)** Risk stratification of the first cohort validation set. **(C)** Risk stratification of the second cohort training set. **(D)** Risk stratification of the second cohort validation set. **(E)** Risk stratification of the third cohort training set. **(F)** Risk stratification of the third cohort validation set.

Each patient is scored according to nomograms and divided into high-risk and low-risk groups. The KM survival curves show that there is a significant difference in survival between high-risk group and low-risk group in the training and validation sets (**Figure 8**). Nomograms are good at identifying cases with different risks of death.

## 4 Discussion

The present study shows that there is a gradient difference in immunotherapy efficacy for MM based on the sunlight exposure patterns. The degree of sunlight exposure is positively correlated with immunotherapy response. Head and neck melanoma has the best curative effect, trunk, arms and legs melanoma is the second and AM is the last. AM does not significantly improve OS in ICIs era. Similar studies have also shown that ICIs is less effective in AM than other CM (27). Lower TMB due to little UV exposure in AM may be the underlying reason (28).

In the present study, during the ICIs era, trunk, arms and legs melanoma has the best prognosis, followed by head and neck melanoma, and AM. However, head and neck melanoma showed the greatest improvement in survival rate and the best ICIs efficacy between the two eras. This contradictory result may be explained by the following reasons: According to epidemiological surveys, head and neck melanoma has a worse prognosis than CM from other body areas (29, 30). Head and neck have rich vascularization and lymphatic drainage, melanoma is prone to metastases and even more likely to develop brain metastases (31, 32). Patients diagnosed MM in the ICIs era may not receive immunotherapy. This may be the reason why survival rate for head and neck melanoma are still lower than other melanoma from a macro-population perspective. However, a relevant study has shown that MM patients with primary site chronically exposed to sunlight (head and neck) had significantly higher OS than other CM after receiving anti-PD-1 (33). The survival disadvantage of head and neck melanoma has been reversed by immunotherapy. But this research did not include patients who did not receive ICIs as a control group. There are still some limitations.

Meanwhile, we took the following measures to ensure the accuracy of the results: Firstly, we used PSM to eliminate the bias caused by other mainstay treatments such as chemotherapy, radiotherapy, and surgery. There is no statistical difference in the frequency of using these treatments between the two eras. Secondly, we try to ensure that the survival difference between the two eras was mainly due to immunotherapy. It is still difficult to eliminate the bias of patients receiving other comorbid treatments, such as hormonal and antibiotics. However, the number of people receiving ICIs therapy is rising every year. According to statistics, the utilization rate of ICIs was 8%-12% of patients from 2004 to 2010, with utilization increasing to 29.7% in 2014, so it is reasonable to assume that immunotherapy is the main reason for the increase of survival rate in ICIs era (34). Lastly, we included patients who did not receive ICIs and objectively compared 3- and 5-year OS rate improvements in two eras.

We observed that female patients with non-acral CM exhibited better survival than male during the ICIs era. It has been widely reported that female diagnosed with CM have a survival advantage compared to male, and this survival advantage still persisted in MM (35–38). The immune system response has sex-related dimorphism which is mainly caused by sex hormones, and female tends to have a more active immune system (39–42). This may explain the female survival advantage. However, we did not find survival difference between male and female in AM. This may indicate an interplay between body sites and sex in terms of survival outcomes. Arce et al. demonstrated that female had better 5-year disease-specific survival (DSS) compared to male patients in cutaneous head and neck melanoma (43). Further research is needed to confirm this hypothesis. Nonetheless, studies have shown that male benefit more from ICIs than female (44–46). Jang et al. showed that female with melanoma have a 2-fold higher mortality risk than their male counterparts in nivolumab plus ipilimumab combination immunotherapy (47). Female mount stronger innate and adaptive immune responses than male on average (48). Tumors in female are more able to evade immune surveillance than male, making tumors less immunogenic and more resistant to immunotherapy (49). The relationship between sex, survival outcome and immunotherapy response is complex and contradictory. The survival gap between male and female MM patients may be narrowed with the widespread use of immunotherapy.

The combination of surgical treatment and ICIs therapy may achieve superior therapeutic effects. We observed that, in non-acral CM, the surgical margin of primary lesion more than 1cm and local lymph node resection combined with immunotherapy show synergistic effects. However, this remains speculative since the number and extent of local lymph node resection are unknown.

Patients showed gradient differences in OS based on sunlight exposure patterns of primary site. Thus, refined categorical management might be needed for MM patients. According to sunlight exposure patterns of primary site, we attempt to create three nomograms to predict 3- and 5-year OS rate. All nomograms showed good discrimination, clinical usability, and risk stratification. This nomogram risk stratification is used to manage patients so that they can receive immunotherapy or combination therapy with different intensities. High-risk patients may consider multidrug immunotherapy, targeted drugs combined with immunotherapy, or immunotherapy combined with radiotherapy and chemotherapy. Lower-intensity treatment options, such as single-agent immunotherapy or single-agent immunotherapy combined with surgery, should be considered in low-risk patients to reduce economical burden and immunotoxicity.

There are still some limitations to our research. We did not directly measure the UV exposure of the skin. Based on previous literature and clinical practice, we divided cohorts according to the amount of sun exposure the skin received, assuming that head and neck were chronically sunlight exposure areas, trunk, arms and legs were intermittent sunlight exposure areas, and acral sites were lesser sunlight exposure areas (12–14). We actually classify cohorts according to sunlight exposure pattern at the anatomical location of the primary tumor. Due to the limitations of the SEER database, we do not have detailed information on the use of ICIs or targeted drugs, comorbidities, and receiving comorbidity-related treatments. The difference in survival between patients in the non-ICIs and ICIs era was used to represent the immunotherapy efficacy. With the development of medical technology, the advancement of non-immunotherapeutic such as surgery, radiotherapy and chemotherapy could also play a more positive role in improving the survival outcome of patients (50).

Nivolumab (a PD-1 checkpoint inhibitor) and ipilimumab (a CTLA-4 checkpoint inhibitor) have complementary activities in MM, compared with ipilimumab monotherapy, two-drug treatment significantly improved the PFS of patients (51, 52). If more information is available on the use of ICIs, we could study the relationship between sunlight exposure patterns in primary site and the efficacy of anti-CTLA-4, anti-PD-1/PD-L1 single- or multiple-agent treatment. This is what we need to study in depth next.

## 5 Conclusion

In summary, based on the classification of sunlight exposure patterns, there is a gradient difference in immunotherapy efficacy for MM. The degree of sunlight exposure is positively correlated with immunotherapy response. Head and neck melanoma has the best curative effect, trunk, arms and legs melanoma is the second and AM is the last. In the ICIs era, age, sex, ulceration, and surgical margin of primary lesion are independent prognostic factors for non-acral CM. M stage and radiotherapy are independent prognostic factors for AM during the ICIs era. The AUC and calibration graphs of the nomogram perform well. The nomograms are of good clinical utility and are internally validated to provide accurate predictions of 3- and 5- year OS rate.

## Data availability statement

The original contributions presented in the study are included in the article/supplementary material. Further inquiries can be directed to the corresponding authors.

## Author contributions

ML and WL conceptualized the study. XM, YC and BW contributed to process data. MC and LZ contributed to validation. SZ, AC, and YP contributed to visualization. JG and JZ administered the project. All authors contributed to the article and approved the submitted version.
